# The formation of estrogen-like tamoxifen metabolites and their influence on enzyme activity and gene expression of ADME genes

**DOI:** 10.1007/s00204-017-2147-y

**Published:** 2017-12-28

**Authors:** Janina Johänning, Patrick Kröner, Maria Thomas, Ulrich M. Zanger, Astrid Nörenberg, Michel Eichelbaum, Matthias Schwab, Hiltrud Brauch, Werner Schroth, Thomas E. Mürdter

**Affiliations:** 1 Dr. Margarete Fischer-Bosch-Institute of Clinical Pharmacology and University of Tübingen, Auerbachstr. 112, 70376 Stuttgart, Germany; 2 Department of Clinical Pharmacology, Institute of Experimental and Clinical Pharmacology, University Hospital, Auf der Morgenstelle 8, 72076 Tübingen, Germany; 3 Department of Pharmacy and Biochemistry, University of Tübingen, Tübingen, Germany; 4 upcyte technologies GmbH, Osterfeldstraße 12-14, 22529 Hamburg, Germany; 5 German Cancer Consortium (DKTK) and German Cancer Research Center (DKFZ), Im Neuenheimer Feld 280, 69120 Heidelberg, Germany

**Keywords:** Tamoxifen, Metabolism, Estrogen-like metabolites, CYP activity, Gene induction

## Abstract

Tamoxifen, a standard therapy for breast cancer, is metabolized to compounds with anti-estrogenic as well as estrogen-like action at the estrogen receptor. Little is known about the formation of estrogen-like metabolites and their biological impact. Thus, we characterized the estrogen-like metabolites tamoxifen bisphenol and metabolite E for their metabolic pathway and their influence on cytochrome P450 activity and ADME gene expression. The formation of tamoxifen bisphenol and metabolite E was studied in human liver microsomes and Supersomes™. Cellular metabolism and impact on CYP enzymes was analyzed in upcyte® hepatocytes. The influence of 5 µM of tamoxifen, anti-estrogenic and estrogen-like metabolites on CYP activity was measured by HPLC MS/MS and on ADME gene expression using RT-PCR analyses. Metabolite E was formed from tamoxifen by CYP2C19, 3A and 1A2 and from desmethyltamoxifen by CYP2D6, 1A2 and 3A. Tamoxifen bisphenol was mainly formed from *(E)*- and *(Z)-*metabolite E by CYP2B6 and CYP2C19, respectively. Regarding phase II metabolism, UGT2B7, 1A8 and 1A3 showed highest activity in glucuronidation of tamoxifen bisphenol and metabolite E. Anti-estrogenic metabolites *(Z)-*4-hydroxytamoxifen, *(Z)-*endoxifen and *(Z)*-norendoxifen inhibited the activity of CYP2C enzymes while tamoxifen bisphenol consistently induced CYPs similar to rifampicin and phenobarbital. On the transcript level, highest induction up to 5.6-fold was observed for CYP3A4 by tamoxifen, *(Z)*-4-hydroxytamoxifen, tamoxifen bisphenol and *(E)*-metabolite E. Estrogen-like tamoxifen metabolites are formed in CYP-dependent reactions and are further metabolized by glucuronidation. The induction of CYP activity by tamoxifen bisphenol and the inhibition of CYP2C enzymes by anti-estrogenic metabolites may lead to drug–drug-interactions.

**Electronic supplementary material:**

The online version of this article (10.1007/s00204-017-2147-y) contains supplementary material, which is available to authorized users.

## Introduction

**Fig. 1:**
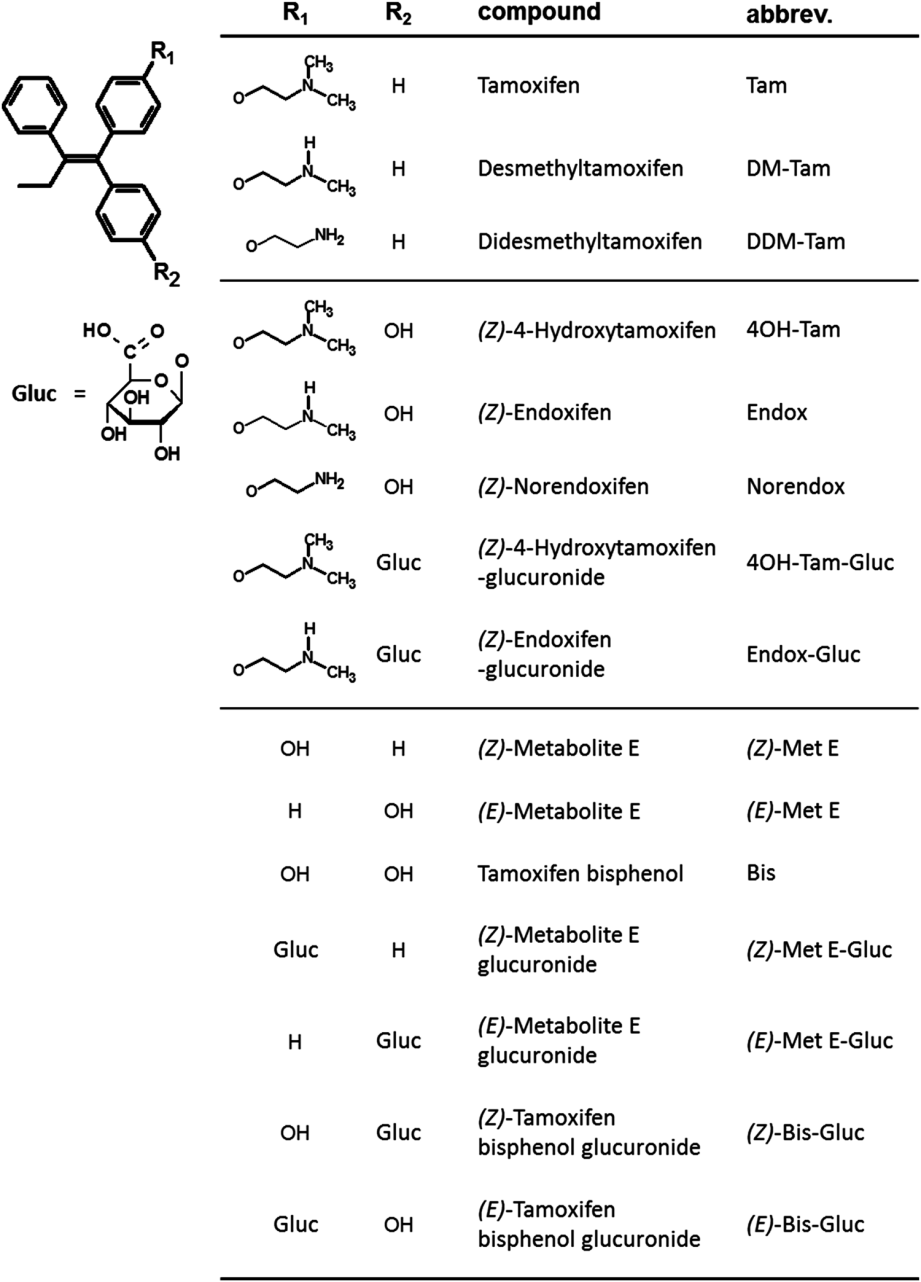
Chemical structures of Tam and its metabolites

Tamoxifen (Tam) is the standard therapy for estrogen receptor (ER)-positive breast cancer in premenopausal women and in men and an alternative to aromatase inhibitors in postmenopausal women. As a selective estrogen receptor modulator (SERM) Tam acts by competitive binding to the ER, which leads to tissue-dependent receptor inhibition or activation, e.g. in breast or bone, respectively (Shang and Brown 2002). Tam is extensively metabolized mostly by enzymes of the cytochrome P450 (CYP) family, resulting in metabolites with anti-estrogenic, as well as estrogen-like properties (Fig. 1) (Jordan et al. 1977; Furr and Jordan 1984; Robinson and Jordan 1988; Wiebe et al. 1992; Desta et al. 2004). *(Z)-*4-Hydroxytamoxifen (4OH-Tam) and *(Z)-*endoxifen (Endox) are the most important anti-estrogenic compounds and mediate the anti-proliferative effect of Tam in breast cancer tissue. In addition, there are many more metabolites with unknown or even estrogen-like properties. Of the latter, tamoxifen bisphenol (4-[1-(4-hydroxyphenyl)-2-phenylbut-1-en-1-yl]phenol, Tam-Bis), *(Z)-* and *(E)-*metabolite E (4-[1,2-diphenylbut-1-en-1-yl]phenol, Met E) were found to be full agonists regarding estrogen-regulated mRNA expression in vitro, leading to cell proliferation in estrogen-dependent breast cancer cell lines (Johnson et al. 1989; Murphy et al. 1990). Noteworthy, these metabolites were identified in a xenograft mouse model of Tam resistance based on MCF7 cell-derived tumors, as well as in human breast cancer tissue from patients with clinical resistance to Tam (Wiebe et al. 1992). Hence, Tam-Bis, *(Z)-* and *(E)-*Met E may hamper Tam therapy success.

While the formation and steady-state plasma concentrations of the major Tam metabolite *N*-desmethyltamoxifen (DM-Tam) and the anti-estrogenic metabolites Endox and 4OH-Tam are known to depend on the activity of certain CYP enzymes, namely 2D6, 2B6, 3A4 and 2C9 (Crewe et al. 2002; Coller 2003; Mürdter et al. 2011; Goetz et al. 2013), the enzymes responsible for the formation of the estrogen-like Tam metabolites are unknown. The strong pharmacological and pharmacogenetic links between the formation of Endox and CYP2D6 phenotype (Stearns et al. 2003; Mürdter et al. 2011) indicate that enzymes predicting metabolite plasma levels in vivo can be reliable biomarkers. Therefore, the identification of CYP isoenzymes responsible for the formation of estrogen-like metabolites may further contribute to the prediction of Tam treatment response and adverse drug reactions. Moreover, drug metabolite interactions that influence the activity of drug-metabolizing enzymes and/or their encoding genes were shown for *N*-didesmethyl-4-hydroxytamoxifen (Norendox) inhibiting various members of the CYP family (Liu et al. 2013), the inhibition of aromatase CYP19A1 by Endox (Lu et al. 2012) and the gene induction of CYP3A4 by Tam and 4OH-Tam (Desai et al. 2002; Sane et al. 2008; Harmsen et al. 2009). Whether estrogen-like Tam metabolites also affect CYP activity or modulate expression of genes involved in absorption, distribution, metabolism and excretion (ADME) of co-subscribed drugs, is unknown.

In this study, the enzymes responsible for the formation of Tam-Bis and Met E were characterized and thereby these metabolites could be integrated in the complex metabolism pathway of Tam for the first time. In addition, the effect of Tam metabolites on the activity and gene expression of ADME enzymes and genes was characterized.

## Methods

### Materials

Human liver microsomes (HLM) were prepared as described (Wolbold et al. 2003) from liver samples of 150 patients undergoing liver surgery at the Campus Virchow, Humboldt University, Berlin, Germany. The study was approved by the local ethics committee of the Charité, Humboldt University Berlin, following the ethical guidelines of the Declaration of Helsinki. Written informed consent was obtained from all patients.

Supersomes™ were obtained from Corning Incorporated, Wiesbaden, Germany, and were specific for CYP isoenzymes CYP1A1, 1A2, 2A6, 2B6, 2C8, 2C9, 2C19, 2D6, 3A4, 3A5 and 19A1. All Supersomes™ contained oxidoreductase. Cytochrome b5 was present in the Supersomes™ of CYP2A6, 2B6, 2C8, 2C9, 3A4 and 3A5. UDP-glucuronosyltransferases (UGT)containing Supersomes™ were specific for the following isoenzymes: 1A1, 1A2, 1A3, 1A4, 1A6, 1A7, 1A8, 1A9, 1A10, 2B4, 2B7, 2B10, 2B15 and 2B17. Upcyte® Hepatocyte growth medium containing supplement A, B and 2 mM l-glutamine, high-performance medium containing supplement A and 2 mM l-glutamine and medium supplements A and B were obtained from upcyte technologies GmbH, Hamburg. l-Glutamine and Dulbecco’s phosphate-buffered saline (DPBS) without calcium or magnesium were purchased from Life Technologies GmbH, Darmstadt. Trypsin/EDTA was obtained from Fisher scientific GmbH, Nidderau. Fetal calf serum (FCS) was purchased from Sigma–Aldrich, Steinheim. Plastic lab ware and collagen type I from rat tail were purchased from BD Biosciences, Heidelberg.

Probe substrates and corresponding internal standards for the measurement of CYP activity were purchased as follows: Phenacetin, amodiaquine, tolbutamide and chloroxazone (Sigma-Aldrich, Steinheim); *S*-mephenytoin and atorvastatin (Toronto Research Chemicals, Toronto); propafenone and 5-OH-propafenone (Knoll, Ludwigshafen); [^2^H_7_]-5-hydroxypropafenone hydrochloride, bupropion hydrochloride, hydroxybupropion hydrochloride, [^2^H_3_]-hydroxybupropion hydrochloride, 4′-hydroxymephenytoin and [^2^H_3_]-4′-hydroxymephenytoin were synthesized in house. For metabolism studies of Tam metabolites, tamoxifen and *(Z)-*4-hydroxytamoxifen were purchased from Sigma-Aldrich, Steinheim and [^2^H_5_]-*N*-desmethyl tamoxifen from Toronto Research Chemicals, Toronto. *N*-desmethyl tamoxifen was a gift from Klinge Pharma GmbH, Munich. *(Z)-*endoxifen (> 98% (Z)), tamoxifen bisphenol, *(E)*- and *(Z)*-metabolite E, glucuronides of 4OH-Tam, Endox, Tam-Bis and both isomers of Met E, [^2^H_5_]-Bisphenol, (E/Z)-[^2^H_5_]-metabolite E, [^2^H_3_]-tamoxifen, *(E)-* and *(Z)-*[^2^H_5_]-4-OH-tamoxifen, *(E)-* and *(Z)-*[^2^H_5_]-Tamoxifen-4-*O*-glucuronide were synthesized in house (Mürdter et al. 2011; Johänning et al. 2015). All stock solutions were stored at − 20 °C. All other chemicals were of analytical grade.

### Cell lines and cultivation

upcyte® hepatocytes 653-03 and 653-03 #138 (653-03 2D6) were obtained from upcyte technologies GmbH, Hamburg. The CYP2D6-expressing subclone #138 was generated by lentiviral transduction as a derivative of the CYP2D6 lacking 653-03 cell line by upcyte technologies GmbH. Cells were cultivated in hepatocyte culture medium at 37 °C and 5 °% CO_2_ in a humidified incubator. Before starting experiments, cells were cultivated in hepatocyte culture medium containing 0.5% (v/v) DMSO (Preculture medium) for 5 days (density of 0.8 × 10^4^ cells/cm^2^) and were subcultivated in high-performance medium containing 0.1% (v/v) DMSO (Metabolism conditioning medium) for 3–5 days (density of 1.6 × 10^5^ cells/cm^2^, confluence). The medium was replaced every 2–3 days. All plastic ware used for cell cultivation was coated with collagen type I. For validation of both cell lines, the base line CYP activity and the induction of CYP enzymes upon treatment with the prototypical inducers rifampicin (Rifa) and phenobarbital (PB) on the level of protein expression and enzymatic activity, as well as the mRNA expression of ADME related genes, were determined (supplement Fig. 1).

### Assessment of metabolic pathways

Supersomes™ (20 pmol CYP enzyme) or pooled human liver microsomes (HLM, 25 µg protein per sample) were pre-incubated in duplicates with 50 µM of substrates (Tam, DM-Tam, DDM-Tam, 4OH-Tam, Endox, Norendox, *(E)*- and *(Z)*-Met E) in 100 mM sodium phosphate buffer (pH 7.4) for 10 min on ice. The reaction was started by adding a NADPH regenerating system (5 mM MgCl_2_, 5 mM glucose-6-phosphate, 0.5 mM NADP^+^ and 10 U glucose-6-phosphate-dehydrogenase in 100 mM sodium phosphate buffer pH 7.4) to the samples and immediately put in a 37 °C water bath for 30 min. The reaction was stopped with the same volume of acetonitrile (ACN) containing 1% of acetic acid together with the corresponding deuterated internal standards. Regarding incubations with Supersomes™ containing UGTs, 5 µg of protein was incubated with 250 mM MgCl_2_ and 2.5 ng of alamethicin for 30 min on ice, followed by 10 min at 37 °C in a water bath. Subsequently, the Supersomes™ were incubated with 10 µM of substrates (Tam-Bis, *(E)*- and *(Z)*-Met E) and the glucuronidation was started by addition of 10 mM of uridine 5′-diphospho-glucuronic acid to the samples in a water bath at 37 °C for 30 min. The reaction was stopped as described above. Metabolites were analyzed by HPLC-ESI-MS/MS.

For the assessment of formation kinetics of Tam-Bis, *(E)*- and *(Z)*-Metabolite E, HLM were incubated with increasing substrate concentrations ranging from 1 to 50 µM as described above. As control for each concentration a sample without NADPH-regenerating system was prepared.

For metabolism studies in ucyte® hepatocytes, the conditioned cells were incubated as triplicates with 1 µM of Tam (control 0.01% DMSO) in high-performance medium for up to 144 h without medium change. The culture medium was collected after 24, 48, 72, 96 and 144 h followed by metabolite measurements using HPLC-ESI-MS/MS.

The influence of Tam metabolites on the activity of CYP enzymes (*n* ≥ 5) and the expression of ADME genes (*n* = 9 for Tam, 4OH-Tam, Endox, Norendox and Tam-Bis; *n* = 3 for Met E) were assessed by incubating cells with 5 µM of the test substances (Tam, 4OH-Tam, Endox, Norendox, Tam-Bis, *(E)*-Met E, *(Z)*-Met E, control 0.05% DMSO) in high-performance medium for 72 h with daily renewal of treatment medium. The induction potency of CYP activity by Tam-Bis was evaluated by incubating cells with increasing concentrations of Tam-Bis ranging from 50 pM to 5 µM.

For analysis of the inhibition potency of 4OH-Tam, Endox and Norendox HLM were pre-incubated for 10 min with increasing concentrations ranging from 200 nM to 10 µM of the substances on ice. CYP activity was determined by the addition of isoenzyme-specific substrates for 15 min in a 37 °C water bath and quantification using HPLC-ESI-MS/MS.

### HPLC-ESI-MS/MS measurement of Tam and its metabolites

For the quantification of Tam and its metabolites from HLM and supersome™ incubations, as well as cell media, previous methods were adapted (Mürdter et al. 2011; Johänning et al. 2015). Protein was precipitated by the addition of ACN containing 1% of acetic acid and the corresponding deuterated internal standards to the same volume of the sample followed by centrifugation at 20.000 g for 15 min at RT. To analyze Tam and its anti-estrogenic metabolites as well as their corresponding glucuronides, the clear supernatant was diluted with the same volume of 0.1% formic acid in water and 20 µl was applied to LC-MS/MS. Analytes were separated on a Zorbax Eclipse Plus C18 column (1.8 µm, 100 × 2.1 mm, Agilent technologies) and detected on a 6460 triple quadrupole mass spectrometer (Agilent technologies) in positive ESI MRM mode. Glucuronides of estrogen-like metabolites were detected separately via MS/MS-measurements in negative ESI MRM mode. For quantification of estrogen-like Tam metabolites proteins were precipitated with 1% of acetic acid in ACN containing the corresponding deuterated internal standards and the supernatant (HLM and supersome™ incubations: 100 µl, cell media: 400 µl) was mixed with 2 volumes of 0.1 M acetic acid and subjected to solid-phase extraction using a C18 Bond Elut© 96-well plate (Agilent Technologies) followed by derivatization and HPLC-MS/MS measurement as described.

### Measurement of CYP activity

Cells were incubated for 3 h at 37 °C with 1 ml of a mixture of probe substrates: 50 µM phenacetin (CYP1A2), 5 µM amodiaquine (CYP2C8), 100 µM tolbutamide (CYP2C9), 100 µM S-mephenytoin (CYP2C19), 25 µM bupropion (CYP2B6), 5 µM propafenone (CYP2D6) and 35 µM atorvastatin (CYP3A4). Metabolites formed were quantified via HPLC-MS/MS as described previously (Feidt et al. 2010).

### Gene expression analysis

Total RNA was isolated from cells using the Direct-zol™ RNA MiniPrep kit (Zymo Research Corp., Irvine, California). cDNA was synthesized using TaqMan Reverse Transcription Reagents (Life technologies, Applera GmbH, Darmstadt, Germany) and the expression of 25 genes representative of phase I and phase II drug metabolism and transport was quantified by Fluidigm’s BioMark HD high-throughput quantitative chip platform (Fluidigm Corporation, San Francisco, USA). For relative gene expression analysis, the delta-delta-Ct (ΔΔCt) method was used: the Ct values of the target mRNA were first normalized to those of the endogenous control glyceraldehyde-3-phosphate dehydrogenase (GAPDH) followed by dividing values of experimental samples by their corresponding DMSO-treated controls to calculate the x-fold changes of mRNA expression.

### Statistics and data analysis

For HPLC-MS/MS measurements, calculation of linear calibration curves and quantification were performed using MassHunter Quantitative Analysis (version B.06.00, Agilent Technologies). Regarding *(E)*- and *(Z)*-Tam-Bis-Gluc, the AUC of the peaks of both isomers were combined for the calculation of the calibration curves and of concentration in the cell media samples. In the case of supersome™ incubations, the isomers of *(E)*- and *(Z)*-Tam-Bis-Gluc were quantified separately.

To account for trace amounts of estrogen-like metabolites present in substrate solutions (in general < 0.01 and 0.15% for Norendox) background substraction of these levels was done in all incubations. In HLM and supersome™ incubations the formation rate is given as nmol µg^−1^ min^−1^ and fmol pmol^−1^ min^−1^, respectively, and was calculated as the mean ± SD. For formation kinetics Vmax and Km was determined via Michealis-Menten kinetic using GraphPad Prism (version 5.04) after background substraction of the corresponding control sample. The intrinsic Clearance (Cl_int_) was given as Vmax/Km ratio. Regarding Tam-Bis formation from hydroxylated Tam metabolites, due to substrate inhibition at higher concentrations Cl_int_ was determined from the initial slope of the curve. Concentrations of Tam and its metabolites in hepatocyte incubations are given as mean values ± SD. Activity of CYP enzymes in induction assays is given as the fold-change (mean ± SD) compared to the corresponding DMSO-treated control. Differences between induced and control samples were compared using Welch’s t-test (GraphPad Prism, version 5.04), when the fold-change was considered biologically relevant (1.5-fold increase or decrease in fold-changes) and corrected for multiple testing according to Bonferroni. Gene expression patterns of treated cells are given as fold-change compared to the corresponding DMSO-treated control samples, as described above. An unpaired t-test assuming unequal variances was used for testing of differences in the expression patterns, when the fold-change was considered biologically relevant (1.5-fold increase or decrease in fold-changes) and corrected using Bonferroni adjustments. In induction and inhibition experiments corresponding EC_50_ and IC_50_ values were calculated by nonlinear regression. In all experiments a level of probability (*p*) ≤ 0.05 for statistical testing was considered significant. The data and statistical analysis comply with the recommendations on experimental design and analysis in pharmacology (Curtis et al. 2015).

## Results

### Identification of the reaction type leading to estrogen-like Tam metabolites from relevant precursors

**Fig. 2:**
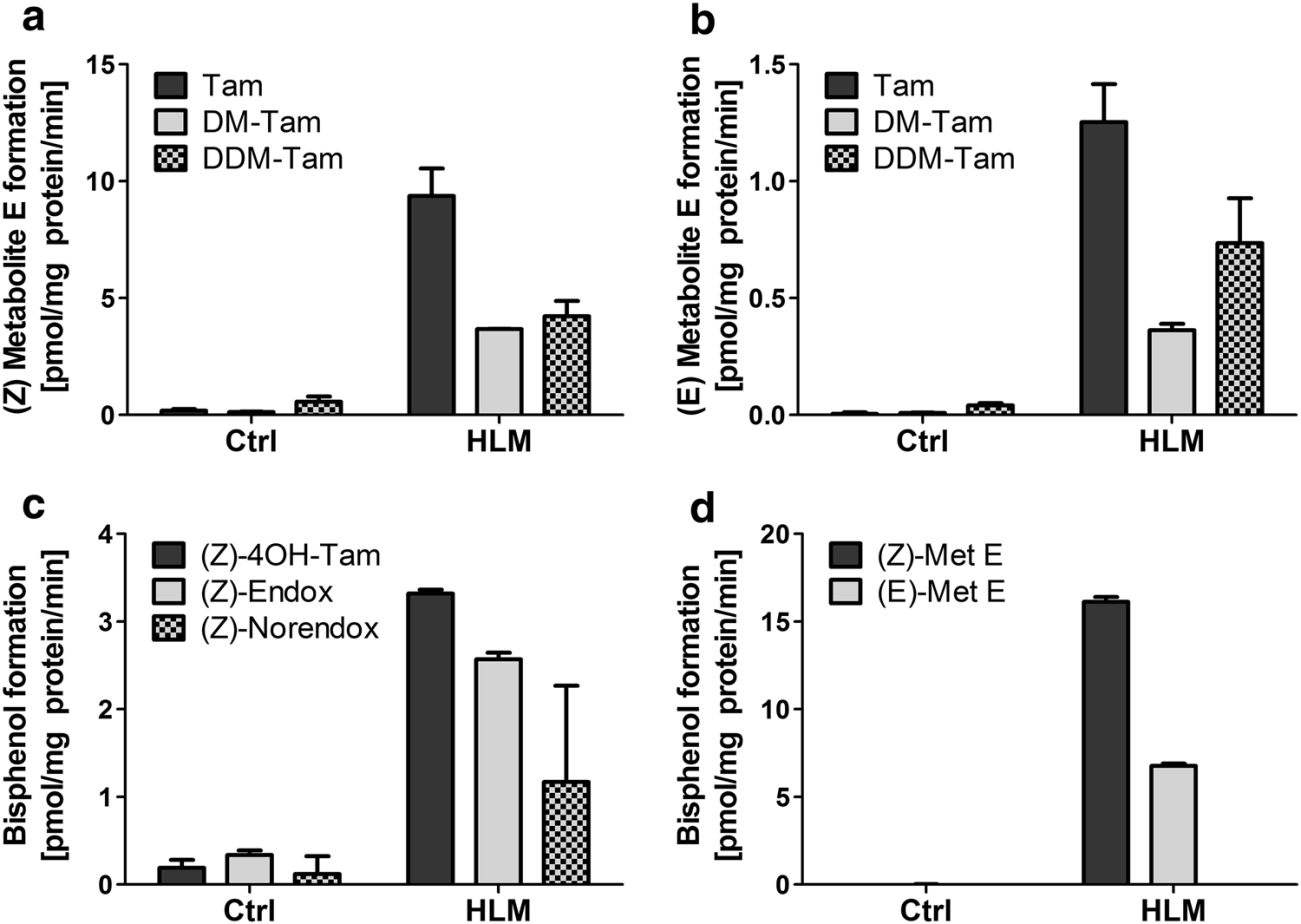
Metabolism of the precursors Tam, DM-Tam and DDM-Tam to Met E and 4OH-Tam, Endox, Norendox and both isomers of Met E to Tam-Bis. Pooled human liver microsomes (HLM) were incubated with 50 µM of Tam, DM-Tam and DDM-Tam to analyze formation to *(Z)*- (**a**) and *(E)*-Met E (**b**) or 50 µM of 4OH-Tam, Endox and Norendox (**c**), as well as both isomers of Met E (**d**) to analyze the metabolism to Tam-Bis. Data are presented as mean ± SD

Human liver microsomes (HLM) were incubated with and without a NADPH-regenerating system to characterize the formation of the estrogen-like Tam metabolites Tam-Bis, *(Z)*- and *(E)*-Met E from Tam and relevant precursors. Both isomers of Met E were formed from Tam, DM-Tam and DDM-Tam with 8-273 times higher formation rates compared to the control, indicating a NADPH-dependent, microsomal reaction. Formation rates for the *(Z)*-isomer were highest for Tam with up to 9.4 pmol mg^−1^ min^−1^, followed by DDM-Tam and DM-Tam with 4 pmol mg^−1^ min^−1^ (Fig. 2a). Likewise, the *(E)*-isomer was metabolized mainly from Tam, followed by DDM-Tam and DM-Tam (Fig. 2b). Of note, formation rates for the *(E)*-isomer were only approximately 10% compared to *(Z)*-Met E. Tam-Bis was also formed in a NADPH-dependent, microsomal reaction from the anti-estrogenic Tam metabolites 4OH-Tam, Endox and Norendox with up to 18 times higher formation rates compared to the control. Here, highest formation rates were observed from 4OH-Tam with 3.3 pmol mg^−1^ min^−1^, followed by Endox and Norendox (Fig. 2c). However, the formation rate of Tam-Bis from Met E was found to be higher than from the anti-estrogenic metabolites: 5–14 times for the *(Z)*-isomer and 2–6 times for the *(E)*-isomer, respectively (Fig. 2d). These NADPH-dependent, microsomal reactions indicate that the side chain cleavage of Tam derived precursors to the estrogen-like metabolites Tam-Bis and Met E is mediated by CYP-enzymes.

### Identification of CYP isoenzymes responsible for the formation of estrogen-like metabolites

**Fig. 3:**
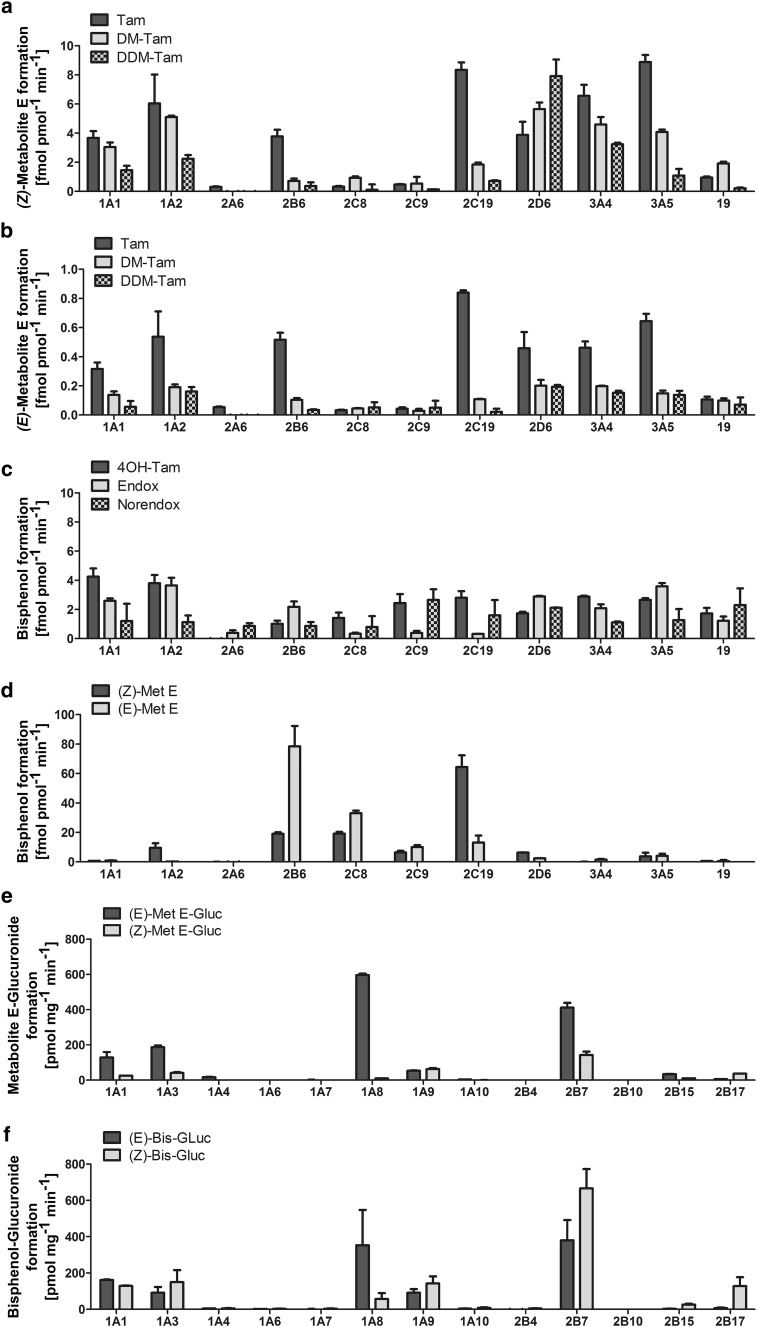
Identification of CYP and UGT enzymes responsible for the metabolism to estrogen-like Tam metabolites Met E and Tam-Bis and their glucuronides. Supersomes™ specific for single CYP enzyme isoenzymes were incubated with 50 µM of the Met E precursors Tam, DM-Tam and DDM-Tam to study the CYP isoenzyme-dependent metabolism to *(Z)*- (**a**) and *(E)*-Met E (**b**). The CYP-specific formation of Tam-Bis was investigated by conversion of 50 µM of 4OH-Tam, Endox and Norendox (**c**), as well as both isomers of Met E (**d**). Furthermore, UGT containing Supersomes™ were used to analyze the glucuronidation of Met E (**e**) and Tam-Bis (**f**). Data are presented as mean ± SD

Supersomes™ specific for single CYP isoenzymes were incubated with 50 µM of relevant Met E and Tam-Bis precursors to further characterize the enzymes involved in their formation. For *(Z)*-Met E highest formation rates were found from Tam by CYP2C19, CYP3A4/5 and 1A2 with up to 8.9 fmol pmol^−1^ min^−1^. CYP2D6, 2B6 and 1A1 showed additional but less pronounced activities in the side chain cleavage of Tam to *(Z)*-Met E (Fig. 3a). When DM-Tam was used as a substrate, highest formation rates to *(Z)*-Met E were obtained by CYP2D6 with 5.6 fmol pmol^−1^ min^−1^, followed by CYP1A2 and CYP3A4/5 with 5.1 and 4.6 fmol∙pmol^−1^ min^−1^, respectively. For the metabolism from DDM-Tam to *(Z)*-Met E, CYP2D6 showed highest activity with 7.9 fmol pmol^−1^ min^−1^, followed by CYP3A4 with 3.2 fmol pmol^−1^ min^−1^ (Fig. 3a).

The pattern of CYP isoenzymes catalyzing the formation of *(E)*-Met E was comparable to that of the *(Z)*-isomer but with approximately 10-times lower formation rates. Accordingly, highest formation rates catalyzed by CYP2C19 were observed using Tam as a substrate. The metabolic rate from DM-Tam and DDM-Tam to *(E)*-Met E did not differ between the main contributors CYP2D6, 3A4 and 1A2 (Fig. 3b).

Tam-Bis was formed from the anti-estrogenic tamoxifen metabolites 4OH-Tam, Endox and Norendox at low rates and without any CYP isoenzyme specificity (Fig. 3c). In contrast, the formation of Tam-Bis from Met E was up to 30-times higher for certain CYP isoenzymes reaching up to 78.4 fmol pmol^−1^ min^−1^ which confirmed HLM-based observations. Specifically, a stereo-specific biotransformation of *(E)*-Met E to Tam-Bis by CYP2B6 and of *(Z)*-Met E to Tam-Bis by CYP2C19 was observed (Fig. 3d).

### Identification of UGT isoenzymes responsible for the glucuronidation of estrogen-like Tam metabolites

Supersomes™ specific for single UGT isoenzymes were incubated with Tam-Bis and both isomers of Met E in order to identify the enzymes responsible for the glucuronidation of these compounds. In the case of Met E, different UGT isoenzymes contributed to the glucuronidation of the *(Z)*- and the *(E)*-isomer, respectively. For the glucuronidation of *(Z)*-Met E highest formation rates were found for UGT2B7 with 143 pmol mg^−1^ min^−1^, followed by 1A9 and 1A3, whereas the *(E)*-isomer was mainly glucuronidated by UGT1A8, 2B7 and 1A3 with 4–14 times higher formation rates as its *(Z)*-isomer (Fig. 3e). UGT2B7 also showed highest formation rates for the glucuronidation of Tam-Bis with up to 666 pmol mg^−1^ min^−1^. Furthermore, the glucuronidation of Tam-Bis led to two Tam-Bis-Gluc isomers. Interestingly, while UGT1A9 and 1A1 catalyzed the glucuronidation to both isomers (*(E)*/*(Z)*-ratio 0.6), UGT1A8 mainly produced the *(E)*-isomer and UGT2B17 the *(Z)*-isomer of Tam-Bis-Gluc (Fig. 3f).

### Formation kinetics of estrogen-like Tam metabolites

To determine the formation kinetics of Tam-Bis, *(Z)*- and *(E)*-Met E from metabolic precursors HLM were incubated with increasing substrate concentrations. Tam showed highest Vmax rates of 9.2 pmol mg^−1^ min^−1^ for *(Z)*-Met E, whereas DDM-Tam showed lowest Km concentrations for the formation to this isomer. Regarding *(E)*-Met E the Km values did not differ between Tam and DM-Tam. The intrinsic Clearance (Cl_int_) for Tam, DM-Tam and DDM-Tam to both isomers of Met E showed highest values for the precursor Tam of 0.112 and 0.049 µl mg^−1^ min^−1^, respectively, followed by DDM-Tam and DM-Tam (Table 1). Of note, Cl_int_ values for *(E)*-Met E were in general 2-times lower than for the *(Z)*-isomer. Regarding Tam-Bis formation, the hydroxylation of *(Z)*-Met E was two times faster than from the *(E)*-isomer (17.2 pmol mg^−1^ min^−1^ compared to 8.1 pmol mg^−1^ min^−1^), whereas Km values were comparable (8.2 µM from *(Z)*- and 10.4 µM from *(E)*-Met E). Therefore, highest Cl_int_ was observed from *(Z)*-Met E with 2.103 µl mg^−1^ min^−1^, exceeding the Cl_int_ from anti-estrogenic Tam metabolites to Tam-Bis up to 40-fold and up to 16-fold from the corresponding *(E)*-isomer (Table 1).

**Table 1:** Formation kinetics of estrogene-like Tam metabolites from metabolical precursors

	Bisphenol formation					*(Z)*-Met E formation			*(E)*-Met E formation		
	4OH-Tam^a^	Endox^a^	Norendox^a^	*(Z)*-Met E	*(E)*-Met E	Tam	DM-Tam	DDM-Tam	Tam	DM-Tam	DDM-Tam
*V*_max_ [pmol mg^−1^ min^−1^]	–	–	–	17.2	8.1	9.2	6.1	5.2	0.57	0.32	0.70
Km [µM]	–	–	–	8.2	10.4	82.3	102.0	55.1	11.5	11.5	20.7
*V*_max_/Km [µl mg^−1^ min^−1^]	0.046	0.033	0.041	2.103	0.780	0.112	0.060	0.095	0.049	0.028	0.034

^a^Because of substrate inhibition at higher concentrations, *V*_max_/Km was determined by the slope of the linear regression

### Tam metabolism in human hepatocyte cell lines

**Fig. 4:**
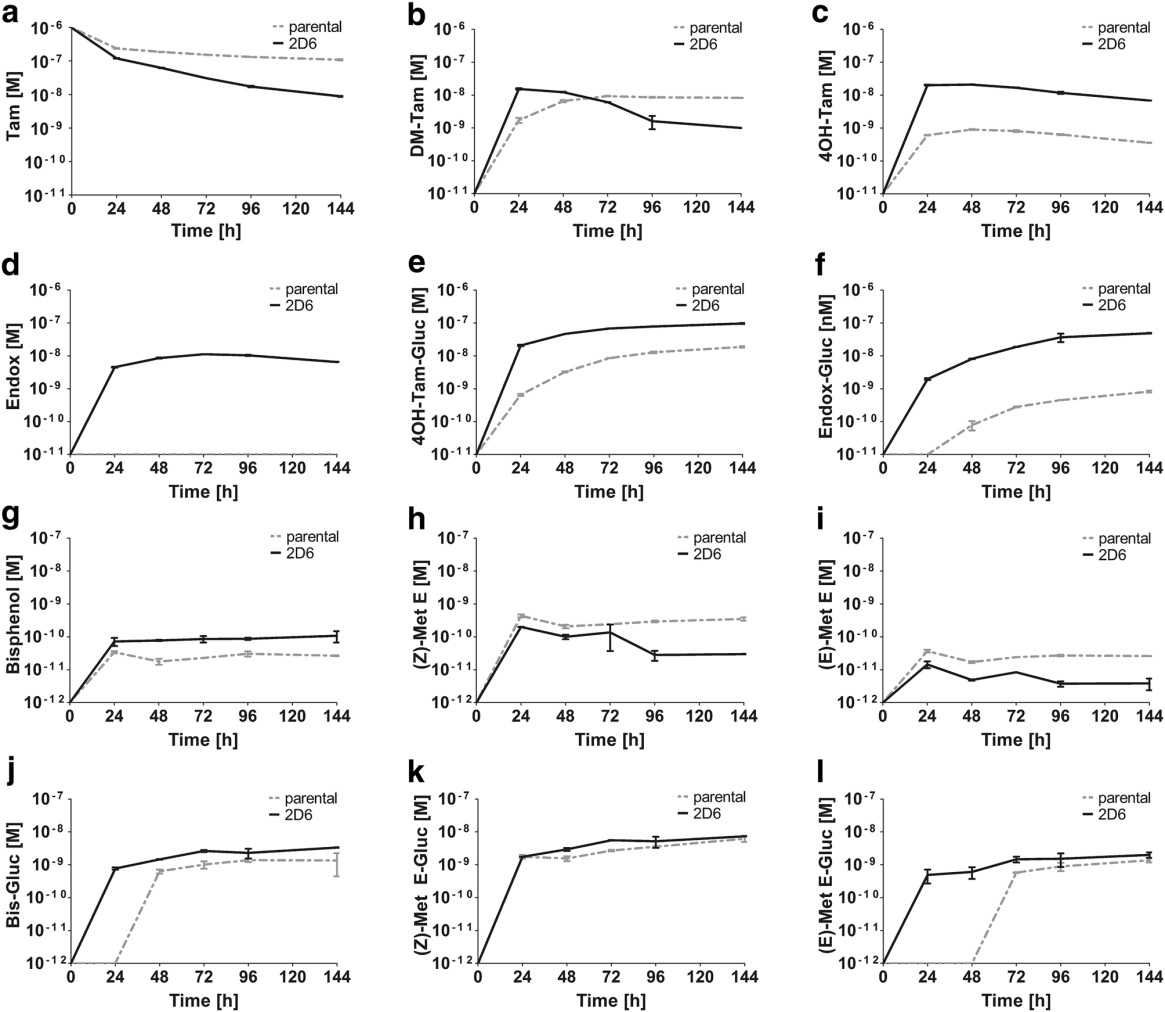
Kinetics of Tam metabolism in human hepatocytes Upcyte® hepatocytes. 653-03 (parental) and 653-03 #138 2D6 (2D6) were incubated with 1 µM of tamoxifen (a) for 6 days. Samples were taken every 24 h and analyzed for phase I metabolites *N*-desmethyl-tamoxifen (DM-Tam) (b), *(Z)*-4-hydroxy-tamoxifen (4OH-Tam) (c), *(Z)*-endoxifen (Endox) (d), tamoxifen bisphenol (Bis) (g), *(Z)*- and *(E)*-metabolite E (Met E) (h and i, respectively) and the respective phase II metabolites tamoxifen-4-*O*-glucuronide (4OH-Tam-Gluc) (e), *N*-desmethyl-tamoxifen-4-*O*-glucuronide (Endox-Gluc) (f), Tam-Bis-glucuronide (Bis-Gluc) (**j**), *(Z)*-Met E-glucuronide ((Z) Met E-Gluc) (**k**) and *(E)*-Met E-glucuronide ((E) Met E-Gluc) (**l**) were quantified in the cell media

To study the Tam metabolism more comprehensively, two strains of upcyte® hepatocytes (653-03 and 653-03 2D6) were used as models for the absence or presence of functional CYP2D6 enzyme activity, resembling opposite phenotypes that are strongly associated with Tam metabolism (Desta et al. 2004; Mürdter et al. 2011). In these cell systems, the metabolic conversion of Tam to DM-Tam, 4OH-Tam, Endox, estrogen-like metabolites and corresponding glucuronides was measured over 6 days. Tam was rapidly taken up in both cell lines in the first 24 h, but 653-03-2D6 cells showed lower Tam concentrations in the culture medium compared to parental cells at later time points (Fig. 4a). The formation of DM-Tam was observed in the culture medium of both, parental and derived cell lines with up to 8 and 14 nM, respectively. Importantly, after 24 h the concentration of DM-Tam decreased in the culture medium of 653-03 2D6 cells, indicating CYP2D6 activity towards hydroxy-metabolite formation (Fig. 4b). In particular, Endox, which is almost exclusively formed from DM-Tam by CYP2D6, was only present in the culture medium of 653-03 2D6 cells with up to 11 nM (Fig. 4d). In contrast, 4OH-Tam was found in the supernatants of both, parental and derived cells but with up to 20 times higher concentrations in the supernatant of 653-03 2D6 cells (Fig. 4c). This proportional difference was maintained in concentrations of the respective glucuronides, i.e. 4OH-Tam-glucuronide was found with up to 25 times higher concentrations in the CYP2D6 expressing cells (Fig. 4e). Although there was no Endox in the supernatant of the parental cell line, Endox-glucuronide was detected at small concentrations in the presence of these cells (Fig. 4f).

With respect to estrogen-like metabolites, highest concentrations with up to 440 pM in the supernatant of the cells were found for *(Z)*-Met E that decreased over time (Fig. 4h). Here, observed concentrations were up to two times higher in the parental cell line. The concentrations of the *(E)*-isomer were, like in previous in vitro findings, at about 10% of those of *(Z)*-Met E at all time points (Fig. 4i). With respect to phase II metabolism, peak glucuronide concentrations were found at 144 h with 7.4 nM for *(Z)*-Met E and 2.0 nM for *(E)*-Met E, thus exceeding the concentrations of their aglycones up to 45-fold in the cell media (Fig. 4k, l). No differences were observed between cell strains. In contrast, Tam-Bis was found in higher concentrations in the supernatant of 653-03 2D6 cells with up to 87.5 pM (30.6 pM in 653-03 cells), which moderately increased over time (Fig. 4g). This approximately threefold difference between cell strains was maintained in phase II metabolism, yet with 30–40-fold higher concentrations for Tam-Bis-glucuronide in both, parental and 2D6-expressing cells (Fig. 4j).

### Influence of Tam and its metabolites on CYP activity and gene expression

**Fig. 5:**
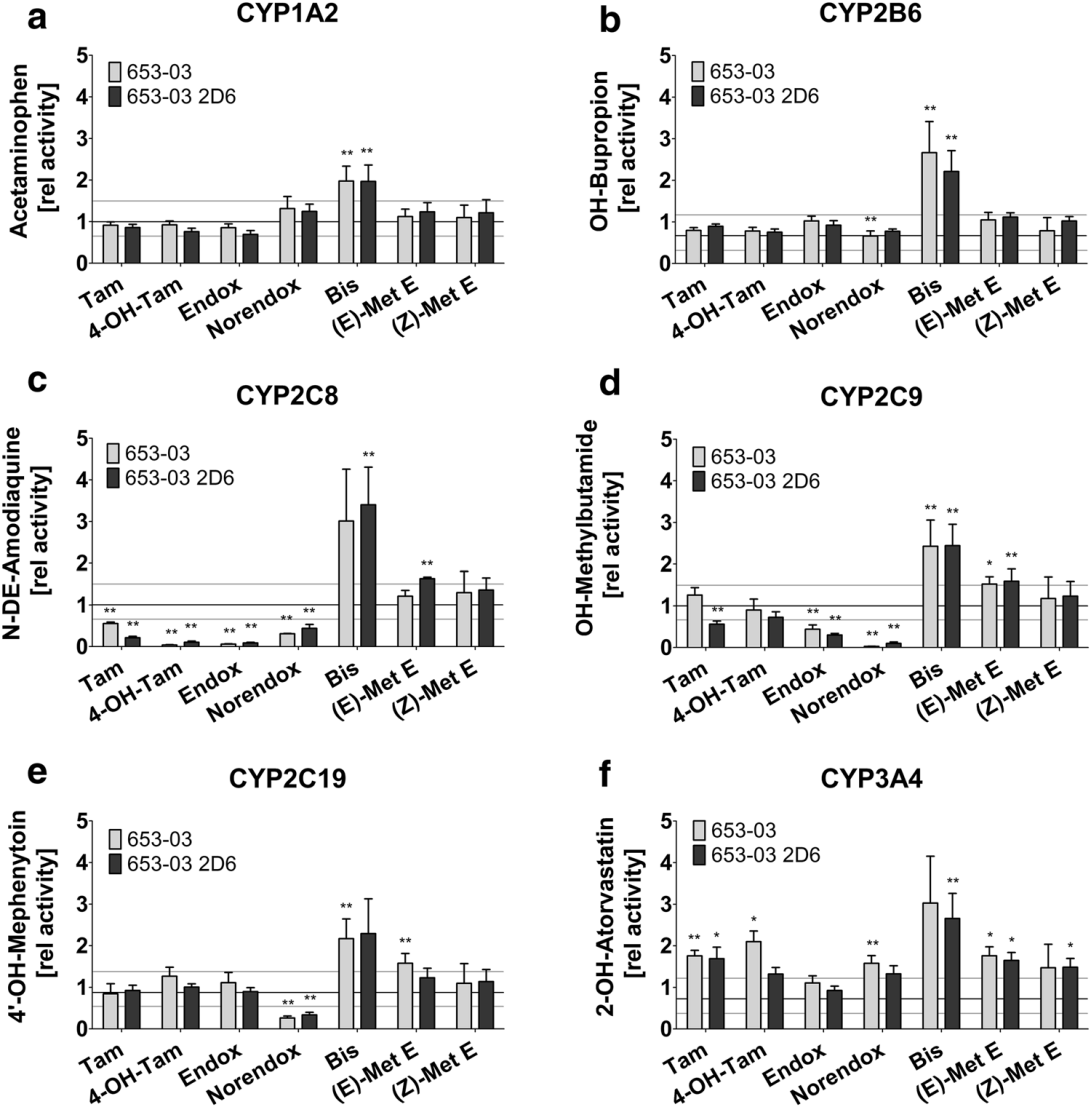
Induction of CYP activity by Tam and anti-estrogenic, as well as estrogen-like metabolites. CYP activity was measured via HPLC-MS/MS after cell treatment for 72 h with 5 µM of tamoxifen (Tam), *(Z)-*4-hydroxytamoxifen (4-OH-Tam), *(Z)-*endoxifen (Endox), *(Z)-*norendoxifen (Norendox), tamoxifen bisphenol (Bis), *(Z)*- and *(E)*-metabolite E (Met E) followed by incubation with model substrates specific for the tested CYP enzymes (**a** CYP1A2; **b** CYP2B6; **c** CYP2C8; **d** CYP2C9; **e** CYP2C19; **j** CYP3A4). Data were normalized to the DMSO treated control and are presented as mean ± SD. *p* values (*p ≤ 0.05; **p ≤ 0.01) were calculated compared to the DMSO treated control and corrected for multiple testing

Previous investigations showed that Tam and its metabolites, including Norendox and 4OH-Tam, are able to modulate the expression and activity of CYP enzymes (Desai et al. 2002). Therefore, these metabolites were included in the analysis of estrogen-like metabolites for interaction effects on CYP enzyme activity and gene expression of ADME genes. A significant induction up to 2.1-fold of CYP3A4 was observed for Tam and 4OH-Tam in both the parental and derived cell line (Fig. 5f). In contrast, Tam and its hydroxylated metabolites strongly inhibited CYP enzymes of the 2C family. Specifically, in both cell lines CYP2C8 was inhibited by up to 90% in the presence of 4OH-Tam and Endox, whereas Tam and Norendox showed modest inhibition (averaged 62.5%) (Fig. 5c). CYP2C9 was inhibited most strongly by Norendox (93%), followed by Endox (63%) (Fig. 5d) and CYP2C19 was solely inhibited by Norendox up to 70% (Fig. 5e). This was reflected in the IC_50_ concentrations using HLMs. Here, Endox and Norendox showed IC_50_ concentrations of 8.1 and 2.4 µM, respectively, for the inhibition of CYP2C8, whereas 4OH-Tam showed only minor inhibition. CYP2C9 was strongly inhibited by Norendox with an IC_50_ of 0.21 µM and weakly inhibited by Endox. Similar to the cell-based observation, Norendox solely inhibited CYP2C19 in HLMs with an IC_50_ concentration of 0.32 µM (Table 2).

**Table 2:** IC50 values of hydroxylated Tam metabolites and EC50 values of Tam-Bis regarding CYP enzyme activity

CYP Isoenzyme	IC50 [µM]			EC50 [nM]		
	4-OH-Tam	Endox	Norendox	Tam-Bis	*(E)*-Met E	*(Z)*-Met E
CYP1A2	n.d	n.d	n.d	518	–	–
CYP2B6	n.d	n.d	n.d	187	–	–
CYP2C8	> 10	8.1	2.4	400	–	–
CYP2C9	–	> 10	0.21	488	–	–
CYP2C19	–	–	0.32	30.5	–	–
CYP3A4	n.d	n.d	n.d	178	277	308

*n.d* not determined

In contrast to the anti-estrogenic metabolites, the estrogen-like Tam-Bis showed no inhibitory effect on either of the tested CYP enzymes, but rather acted as a strong inducer on all tested CYP isoenzymes (Fig. 5) with increasing strength of induction ranging between 2.0- and 3.8-fold as follows: CYP1A2, 2C19, 2C9, 2B6, 3A4 and 2C8 at 5 µM. Here, lowest EC_50_ concentrations were found for the induction of CYP2C19 (30.5 nM) followed by CYP3A4 (178 nM) and CYP2B6 (187 nM). For the remaining CYP enzymes CYP2C8, 2C9 and 1A2 EC_50_ concentrations above 400 nM were observed (Table 2). Likewise, both isomers of Met E did not act as CYP inhibitors, but weakly induced CYP2C9 and CYP3A4 (*E*) and CYP3A4 (*Z*) with similar EC_50_ potency for CYP3A4 of 308 and 277 nM for *(Z)*- and *(E)*-Met E, respectively (Fig. 5d, f; Table 2).

On the level of gene expression, a number of ADME genes were differently expressed upon exposure to Tam or 4-hydroxylated anti-estrogenic metabolites; however, only those genes that were significantly regulated in both cell strains were considered for evaluation: In accordance with the induction of enzyme activity by Tam and 4OH-Tam, CYP3A4 gene expression was most strongly upregulated up to 5.6-fold upon 4OH-Tam treatment (supplement Fig. 2). Furthermore, the expression of CYP1A1 was induced up to 2.3-fold by Endox. In contrast, downregulation was observed for CYP2A6 and transporter gene SLC10A1 by 4OH-Tam up to twofold (supplement Fig.2). With respect to estrogen-like Tam metabolites, the most frequent expression alterations of ADME genes in both cell strains were observed upon Tam-Bis treatment: CYP3A4, CYP1A1 and efflux transporter ABCG2 were induced up to 4.5-fold, whereas expression levels of solute carrier family transporters SLC10A1 and SLC22A7 were up to 2.5-fold lower. For Met E, CYP1A1 expression was upregulated 3.3-fold by *(Z)*-Met E. Although some additional ADME genes were upregulated following Met E isomer or differently regulated upon anti-estrogenic treatment, data were not consistent among both cell lines and therefore remain elusive.

## Discussion

The characterization of the pharmacokinetics of extensively metabolized drugs such as Tam provides critical information to understand the variability of clinical responses and moreover gives insights on possible mechanisms of drug–drug-interactions. This has been demonstrated by several studies showing that polymorphisms of CYP2D6 predict variable bioactivation of Tam to its major anti-estrogenic metabolite *(Z)*-Endox and, therefore, impacts clinical outcome (Schroth et al. 2009; Madlensky et al. 2011; Goetz et al. 2013; Province et al. 2014; Saladores et al. 2015). In contrast, Tam-Bis, *(Z)*- and *(E)*-Met E show full estrogen-like properties by activating ER in breast cells (Johnson et al. 1989) and may hamper therapeutic response accordingly (Wiebe et al. 1992; Osborne and Fuqua 1994). This study aimed to elucidate the metabolism of Tam to estrogen-like metabolites in order to identify determinants of their formation, as well as to identify possible drug–drug-interactions resulting from the complex spectrum of Tam metabolites.

For the first time, it could be shown that the side-chain cleavage of Tam and derived compounds is an active, enzyme-dependent reaction. Furthermore, these data indicate that Tam directly acts as a precursor of Met E, as its Cl_int_ is approximately 2-times higher compared to the two other precursor candidates DM-Tam and DDM-Tam, respectively. Nevertheless, due to the high plasma concentration of DM-Tam in vivo, the formation of Met E most likely depends on the conversion of both, Tam and DM-Tam, whereas the metabolism of DDM-Tam likely plays a minor role. Although the formation of Tam-Bis by hydroxylation of Met E appears to be more favorable given its 40-times faster Cl_int_ compared to side-chain cleavage of hydroxylated Tam metabolites, the formation of Tam-Bis is likely also contributed by the anti-estrogenic precursors 4OH-Tam and Endox, as their concentrations are at least an order of magnitude higher compared to Met E (Johänning et al. 2015).

**Fig. 6:**
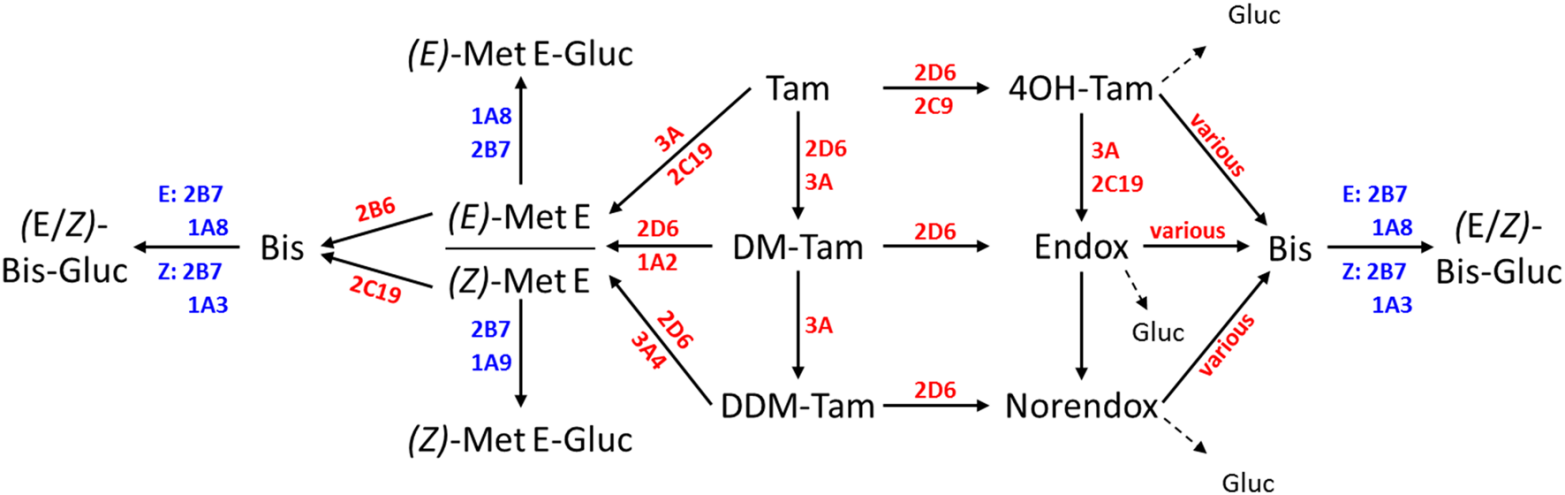
Proposed metabolism scheme of Tam to demethylated, anti-estrogenic and estrogen-like metabolites, as well as corresponding glucuronides. Based on the in vitro data of this study a metabolism scheme of tamoxifen (Tam) to demethylated metabolites *N*-desmethyl-tamoxifen (DM-Tam) and *N*-didesmethyl-tamoxifen (DDM-Tam), anti-estrogenic metabolites *(Z)*-4-hydroxy-tamoxifen (4OH-Tam), *(Z)*-endoxifen (Endox) and *(Z)*-norendoxifen (Norendox), estrogen-like metabolites tamoxifen bisphenol (Bis), *(Z)*- and *(E)*-metabolite E (Met E), as well as corresponding glucuronides (Gluc) including cytochrome P450 enzymes (CYP) and Uridine-5′-diphospho-glucuronosyltransferases (UGT) is suggested

Next, the CYP enzymes responsible for the formation of the estrogen-like Tam metabolites Met E and Tam-Bis were identified for the first time. Met E formation is catalyzed by CYP3A4, CYP1A isoenzymes and CYP2D6 in similar rates from both Tam and DM-Tam, whereas CYP2C19 and CYP2B6 catalyze its formation exclusively from Tam. Given that all Met E catalyzing enzymes are highly polymorphic (Zanger and Schwab 2013), future studies for an association between in vivo plasma concentrations in breast cancer patients and genetic determinants predictive of enzyme function bear potential to establish genetic predictors that may be tested for an association with clinical outcome. Regarding the formation of Tam-Bis, there was no clear substrate specificity across hydroxylated Tam metabolite precursors. Moreover, because various CYP enzymes contributed to the formation of Tam-Bis, a pharmacogenetic impact to explain variability in plasma concentrations is unlikely. Of note, the UGT isoenzymes responsible for the glucuronidation of Tam-Bis and Met E are expressed in various tissues including liver (UGT1A3, 2B7), kidney and urinary bladder (UGT1A8, 1A9) and are characterized by genetic variation (Stingl et al. 2014). Therefore, germline polymorphisms or tissue-specific characteristics including renal failure may influence patients’ exposures to estrogen-like Tam metabolites via impaired phase II elimination with a possible impact on therapy outcome. In summary, these findings led to the proposed incorporation of the estrogen-like Tam metabolites in the complex metabolism pathway of Tam (Fig. 6).

The time course of estrogen-like metabolite formation and corresponding glucuronides was investigated using upcyte® hepatocytes as a model. Given that the formation of clinically relevant Tam metabolites (4OH-Tam and Endox) strongly depend on the CYP2D6 phenotype which may also impact the formation of estrogen-like metabolites, hepatocyte cells representative of a poor metabolizer phenotype and their CYP2D6 expressing derivatives (extensive metabolizer) were used. As expected, DM-Tam was observed as the primary metabolite which was subsequently hydroxylated. Moreover, a strong CYP2D6 dependency of Endox could be confirmed as this metabolite was only detected in the supernatant of the 653-03 2D6 cells. Noteworthy, Endox-glucuronide was found in small concentrations in the presence of 653-03 cells confirming the relevance of a minor contribution of Endox formation via CYP2D6-independent pathways from 4OH-Tam (Dickschen et al. 2012; Heine et al. 2014).

With respect to the estrogen-like Tam metabolites, all three compounds were detected in the culture medium of both, parental and CYP2D6 expressing cells. Tam-Bis was found at up to 4 times higher concentrations in the 653-03 2D6 cells, suggesting that Tam-Bis derives not only from Met E but also from 4-hydroxylated Tam metabolites, confirming our HLM and Supersome™ data. Of note, the metabolism to Met E and the ratio of the *(E)*- to *(Z)*-isomer were independent of the CYP2D6 phenotype, which is also in line with our in vitro data. With respect to phase II metabolism, the concentrations of the glucuronides of Tam-Bis and Met E were more than 10-times higher compared to the unconjugated compounds. Thus, glucuronidation appears to be an important clearance path for estrogen-like metabolites confirming previous data (Lien et al. 1989; Poon et al. 1993). Of note, the metabolic ratio of *(E)*-Met E to its glucuronide was smaller compared to the respective ratio of the *(Z)*-isomers indicating a faster glucuronidation of the *(E)*-isomer, a finding that resembles data for the *(E)*-isomers of 4OH-Tam and Endox (Sun et al. 2006).

Drug interactions due to metabolites influencing the activity of drug metabolizing enzymes and transporters can cause changes in the absorption, metabolism, or excretion of a parent and/or co-administered drug but may also have an influence on endogenous metabolism. Liu and colleagues showed that Norendox inhibited CYP2C19 and aromatase (CYP19A1) activity (Liu et al. 2013), while CYP19A1 activity was also inhibited by Endox (Lu et al. 2012) pointing to a dualistic inhibitory effect of Endox on estrogen-driven breast cancer cells. Furthermore, Tam and 4OH-Tam were shown to induce CYP3A4 mRNA expression (Sane et al. 2008). Here for the first time, estrogen-like Tam metabolites were included in the assessment of interactions that may modulate the activity of CYP isoenzymes and the expression of ADME related genes. While the induction of CYP3A4 enzyme activity by Tam and 4OH-Tam was confirmed (Sane et al. 2008), estrogen-like Tam metabolite Tam-Bis induced the activity of all tested CYP enzymes to an extent similar to rifampicin and phenobarbital while Met E isomers had only moderate inducing capacity on CYP3A4 and CYP2C9. Importantly, since EC_50_ concentrations estimated in cell experiments were higher than the plasma concentrations observed in Tam-treated patients, a plasma-to-tissue gradient as was previously noted for Tam metabolites (Lien et al. 1989, 1991; Kisanga et al. 2004) likely results in an accumulation of estrogen-like Tam metabolites to exert their CYP-inducing effect in vivo. Of note, estradiol itself did not have an impact on CYP activities (supplement Fig. 3), indicating that the CYP-inducing effect of estrogen-like metabolites is independent of their estrogen-like features. Upregulation of CYP3A4 expression upon Tam, 4OH-Tam, Tam-Bis and *(E)*-Met E stimulation suggests that these Tam metabolites act through PXR-activated induction of gene regulation (Sane et al. 2008). Thus, inductions of CYP enzymes, especially CYP3A4, may influence the plasma levels of co-prescribed drugs, an interaction which was observed in the past when aromatase inhibitors like letrozol were used in combination with Tam (Dowsett 1999).

Furthermore, an inhibitory effect of 4OH-Tam, Endox and Norendox on members of the CYP2C family was observed at concentrations which, however, require a tissue enrichment of these Tam metabolites to exert in vivo inhibition as postulated previously (Lien et al. 1989, 1991; Kisanga et al. 2004). Yet, the inhibitory effect was not seen on the level of mRNA. Therefore, either a competitive mechanism as was suggested for the CYP2C19/Norendox interaction (Liu et al. 2013) or an allosteric interaction of Endox and Norendox, as was reported for the aromatase (Lu et al. 2012) may explain the inhibition of CYP2C isoenzymes by hydroxylated Tam metabolites. Interestingly, the estrogen-like Tam metabolites did not show inhibitory effects on the activity of CYP enzymes. These data support the hypothesis that the amine containing side chain of Tam metabolites may provide structural surfaces for CYP enzyme inhibition. In particular, whereas Norendox inhibited all three CYP2C members, 4OH-Tam solely inhibited CYP2C8, pointing to a role of methylation status of the side chain amine in mediating this inhibitory capacity.

Importantly, since 10% of all drugs including cytostatics, anticoagulants and proton pump inhibitors are metabolized by CYP2C enzymes, care must be taken when treating comorbidities with CYP2C substrates in order to avoid toxicities during long-term Tam treatment. Whether the inhibitory effects of anti-estrogenic metabolites and Tam-Bis on SLC-transporter gene expression interfere with the metabolism and distribution of other xenobiotics remains to be elucidated. Yet, our data clearly indicate that anti-estrogenic and estrogen-like Tam metabolites are not only different at the ER (Jordan and Gosden 1982), but may also explain so far overlooked effects on the modulation of other proteins and receptors.

In summary, the metabolic pathways leading to the estrogen-like Tam metabolites Tam-Bis, *(Z)*- and *(E)*-Met E were identified for the first time and integrated into the complex metabolism of Tam (Fig. 6). The role of CYP2D6 as the most critical determinant for Endox had minor influence on the formation of estrogen-like metabolites. High levels of glucuronides compared to the unconjugated compounds point to glucuronidation as a major clearance path for estrogen-like Tam metabolites. The findings of a strong inhibition of the CYP2C family by anti-estrogenic Tam metabolites, as well as the induction of CYP3A4 by both anti-estrogenic and estrogen-like metabolites, indicate the risk of drug–drug-interactions during long-term Tam therapy. The clinical effects of estrogen-like metabolites and their utility as plasma biomarkers to predict adverse reactions and therapy outcome in breast cancer patients need to be demonstrated in clinical investigations.

## Electronic supplementary material

Below is the link to the electronic supplementary material.

Supplementary material 1 (DOCX 15 KB)

Supplementary material 2 (PNG 3687 KB)

Supplementary material 3 (PNG 42 KB)

Supplementary material 4 (PNG 119 KB)
