# Supplementary material for: The formation of estrogen-like tamoxifen metabolites and their influence on enzyme activity and gene expression of ADME genes
Source: Arch Toxicol. 2017 Dec 28;92(3):1099–112. doi: 10.1007/s00204-017-2147-y (PMC5866846; doi:10.1007/s00204-017-2147-y)
Supplement: Supplementary file 1 — Supplementary material 1 (DOCX 15 KB) [file 204_2017_2147_MOESM1_ESM.docx]

**Supplement**

**Material and Methods**

**Induction Assay with prototypical inducers**

Conditioned cells were incubated with the prototypical inducers rifampicin (30 µM, Merck Millipore, Darmstadt) and phenobarbital (2 mM, Sigma-Aldrich, Steinheim) in HPM for 72 h with daily treatment replacement. As control 0.05 % DMSO was used. The basal CYP activity and induction of CYP activity compared to the DMSO control were measured via HPLC-ESI-MS/MS.

**Western Blot**

For western blot analysis cells were seeded in a 24-well plate as described, harvested and lysed using lysis buffer (50 mM Tris pH 7.6, 0.25 M NaCl, 0.1 % Triton x100, 5 mM EDTA, Protease Inhibitor Cocktail (Roche, Penzberg)). 30 µl of the cell lysate were separated on a 10 % polyacrylamide gel via gelelectrophoresis. Proteins were transferred to a PVDF-membrane using semidry blotting. The membrane was subsequently incubated in 5 % skim milk powder in tris buffered saline pH 7.6 with 0.1 % Tween 20. The following antibodies were used: anti-human CYP3A4 (WB-MAB-3A, mouse monoclonal, BD Biosciences, USA), anti-human CYP2D6 (WB-MAB-2D6, mouse monoclonal, Corning Incorporated, USA), monoclonal anti-β-actin (clone AC-74, mouse monoclonal, Sigma Aldrich, USA) and horse anti-mouse antibody linked to horse-reddish-peroxidase (HRP-linked, Cell Signaling Technology, USA). For chemiluminescence detection SuperSignal® West Dura Extended Duration Substrate (Thermo Fisher Scientific Inc., USA) was used and the signal was recorded with a CCD camera.

**Results**

**Expression of CYP enzymes, induction of enzyme activity and ADME gene expression by rifampicin and phenobarbital in upcyte® hepatocytes**

For validation of the upcyte® hepatocytes 653-03 and 653-03 2D6 for their use as hepatic models, the expression, activity and inducibility of CYP enzymes were analyzed via western blot (WB) and CYP activity assay at baseline and upon Rifa and PB incubations.

Protein expression of CYP3A4 was detectable at baseline level and was induced upon Rifa and PB treatment in both cell strains, whereas CYP2D6 could, as expected, only be detected in 653-03 2D6 cells at high levels without indication of an induction (supplement Fig. 1A). The difference of the CYP activity in both cell strains was nearly 50-fold for CYP2D6, whereas the difference of the activity of CYP1A2, CYP2B6, CYP2C8 and CYP2C19 was less than 2-fold and for CYP3A4 and CYP2C9 2.5-fold and 2.8-fold, respectively. In both cell strains Rifa induced CYP2C8, 2C9 2C19, and CYP3A4, while CYP1A2 and 2B6 were not induced (supplement Fig. 1d). PB induced the activity of CYP2B6 and 3A4 in both cell lines (supplement Fig. 1d). These findings were confirmed via gene expression analysis: strongest Rifa-mediated induction of 20- and 12-fold was observed for CYP3A4 in the parental and 2D6 cells, respectively, followed by CYP2C8 and 2C19 (1.6 to 2.4-fold). Furthermore, Rifa treatment induced phase II enzyme GSTA2 by 2.6-fold and ATP-binding cassette transporter ABCB1 by 1.7-fold. In contrast, the CYP enzymes 1A2 and 2E1 were down regulated in the Rifa treated cells to 0.1- and 0.3-fold, respectively, as were phase II enzymes SULT1B1 up to 0.4-fold and NAT2 to 0.5-fold (supplement Fig. 1c). The treatment with PB generated similar gene expression patterns: the expression of CYP2C19 and 3A4 were induced by up to 1.5- and 9-fold, respectively. Likewise, the expression of GSTA2 and UGT1A1 were induced (6 to 8-fold for GSTA2 and 1.8-fold for UGT1A1) in both strains. The gene-expression of transporters ABCB1 and ABCG1 were induced 1.5- to 3-fold and of solute carrier (SLC) SLCO1B1 up to 2.9-fold by PB. On the other hand, PB treatment decreased the expression of CYP2C8 and 2E1 up to 0.3- and 0.2-fold, respectively. The phase II enzymes SULT1B1 and NAT1 were also down regulated by up to 0.5-fold, as was the transporter SLC22A7.

In summary, the upcyte® hepatocytes 653-03 and 653-03 2D6 had reasonably high CYP enzyme activities and responded to prototypical inducers Rifa and PB similar to PHHs, with only slight differences between cell strains, indicating their suitability for metabolism studies.

**Supplement Fig. 1 Characterization of the upcyte® hepatocytes 653-03 and 653-03 2D6 via (a) Western Blot for basal and induced protein expression of the CYP enzymes CYP3A4 and 2D6, (b) basal CYP activity measurements, (c) induced gene expression analysis of different ADME genes and (d) basal and induced CYP activity measurement. The cells were treated with 30 µM rifampicin (Rifa), 2 mM phenobarbital (PB) or corresponding DMSO concentrations as controls for 3 days in all induction experiments. Expression of CYP enzymes on protein level was determined via Western Blot with antibodies for detection CYP3A4 and CYP2D6 and on gene expression level via fluidigm assays.** The heatmap shows a subset of 25 genes representing pathways relevant for drug metabolism and transport. Red and blue colors refer to up- and down regulation compared to DMSO control, white indicates no difference (maximum red intensity = 5-fold up, maximum blue intensity =0.2-fold down). **CYP activity was measured via HPLC-MS/MS after cell treatment followed by incubation with model substrates specific for the tested CYP enzymes. Data are represented as (B) total activity or (D) fold-induction relative to non-induced DMSO control**

**Supplement Fig. 2 Influence of Tam, its anti-estrogenic and estrogen-like metabolites on mRNA expression of ADME related genes** Gene expression was analyzed via fluidigm assay in upcyte® hepatocytes 653-03 and 653-03 2D6 after cell treatment for 72 h with 5 µM of tamoxifen (Tam), (Z)-4-hydroxytamoxifen (4-OH-Tam), (Z)-endoxifen (Endox), (Z)-norendoxifen (Norendox), bisphenol (Bis), (Z)-, and (E)-metabolite E (Met E). The heatmap shows a subset of 25 genes representing pathways relevant for drug metabolism and transport. Red and blue colors refer to up- and down regulation compared to DMSO control, white indicates no difference (maximum red intensity = 5-fold up, maximum blue intensity =0.2-fold down). P-values (* = p≤0.05; ** = p≤ 0.01) were calculated compared to the DMSO treated control and corrected for multiple testing.

**Supplement Fig. 3 Influence of estradiol on the activity of CYP enzymes** Upcyte® hepatocytes 653-03 and 653-03 2D6 were incubated as duplicates with 500 nM Estradiol (E2) or DMSO (control) for 3 days**. CYP activity was measured via HPLC-MS/MS after cell treatment followed by incubation with model substrates specific for the tested CYP enzymes. Data are represented as fold-induction relative to non-induced DMSO control.**
