# Supplementary figures and images for: The formation of estrogen-like tamoxifen metabolites and their influence on enzyme activity and gene expression of ADME genes
Source: Arch Toxicol. 2017 Dec 28;92(3):1099–112. doi: 10.1007/s00204-017-2147-y (PMC5866846; doi:10.1007/s00204-017-2147-y)

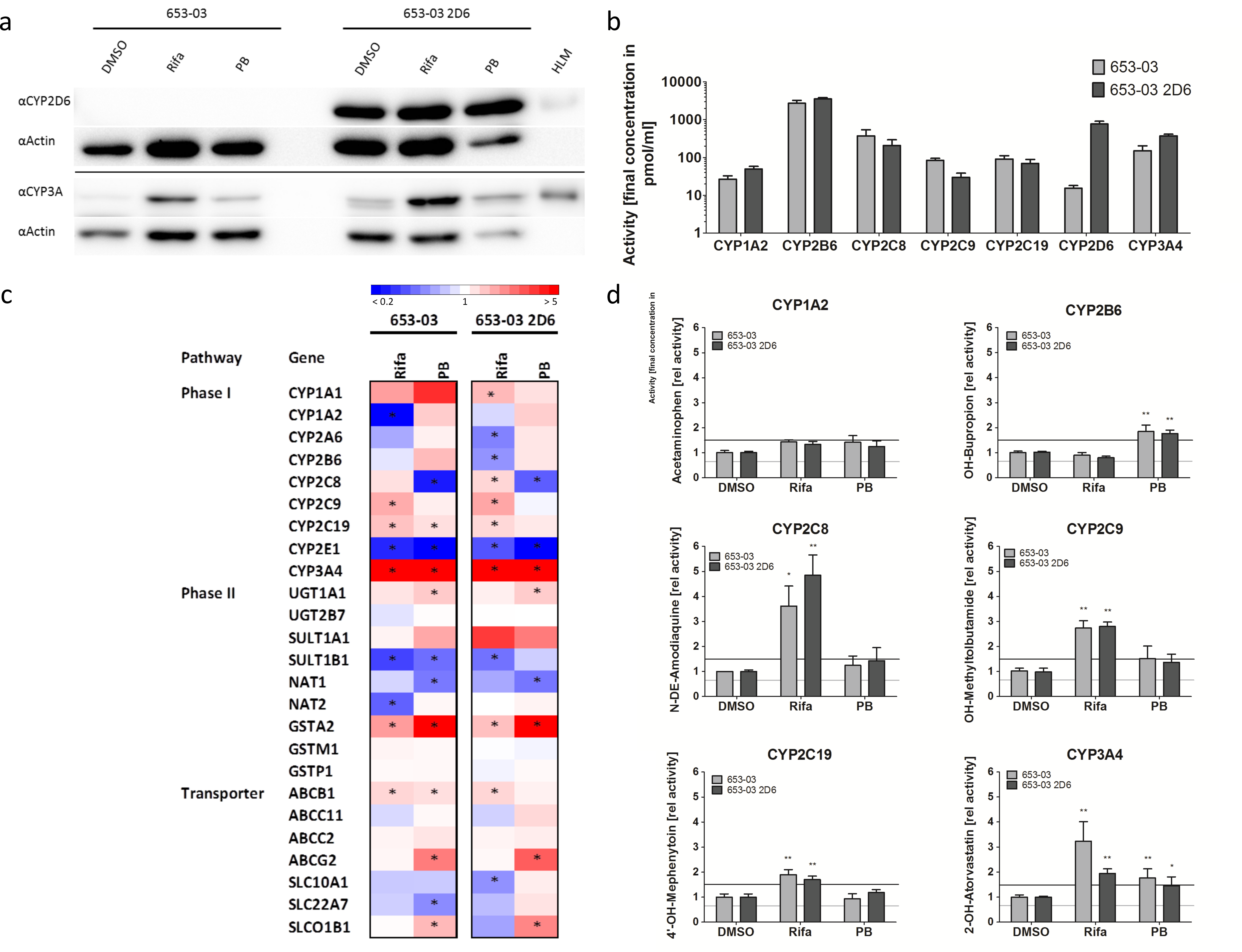

Supplement: Supplementary file 2 — Supplementary material 2 (PNG 3687 KB) [file 204_2017_2147_MOESM2_ESM.png]

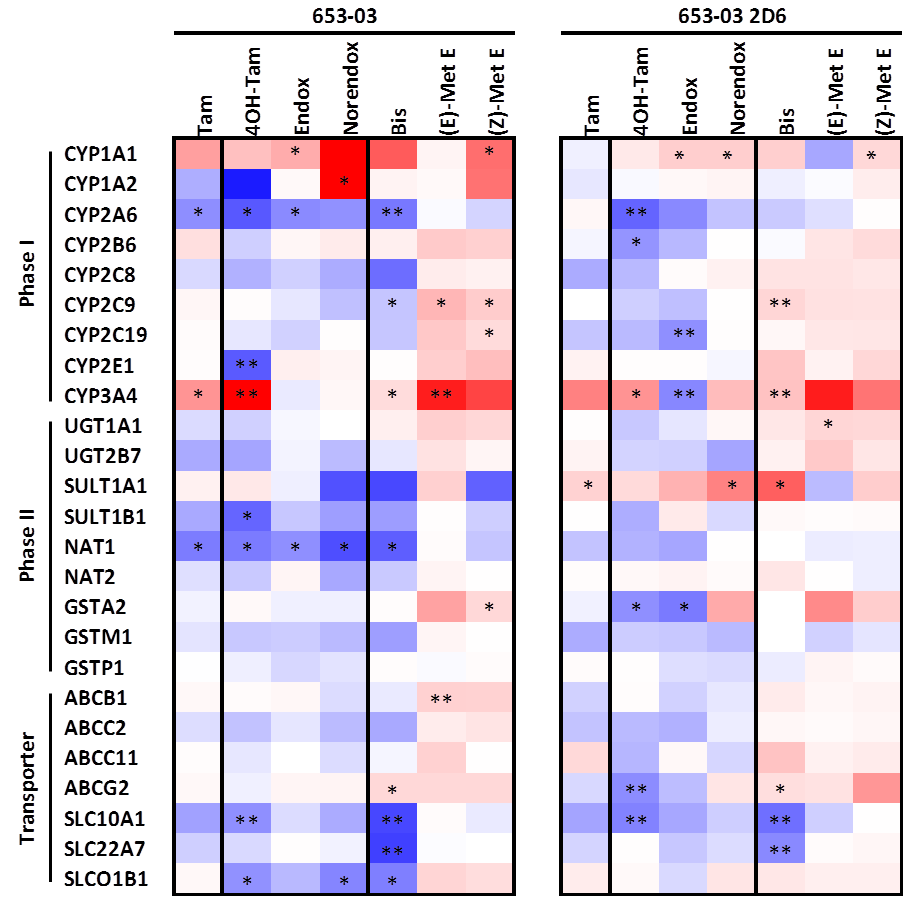

Supplement: Supplementary file 3 — Supplementary material 3 (PNG 42 KB) [file 204_2017_2147_MOESM3_ESM.png]

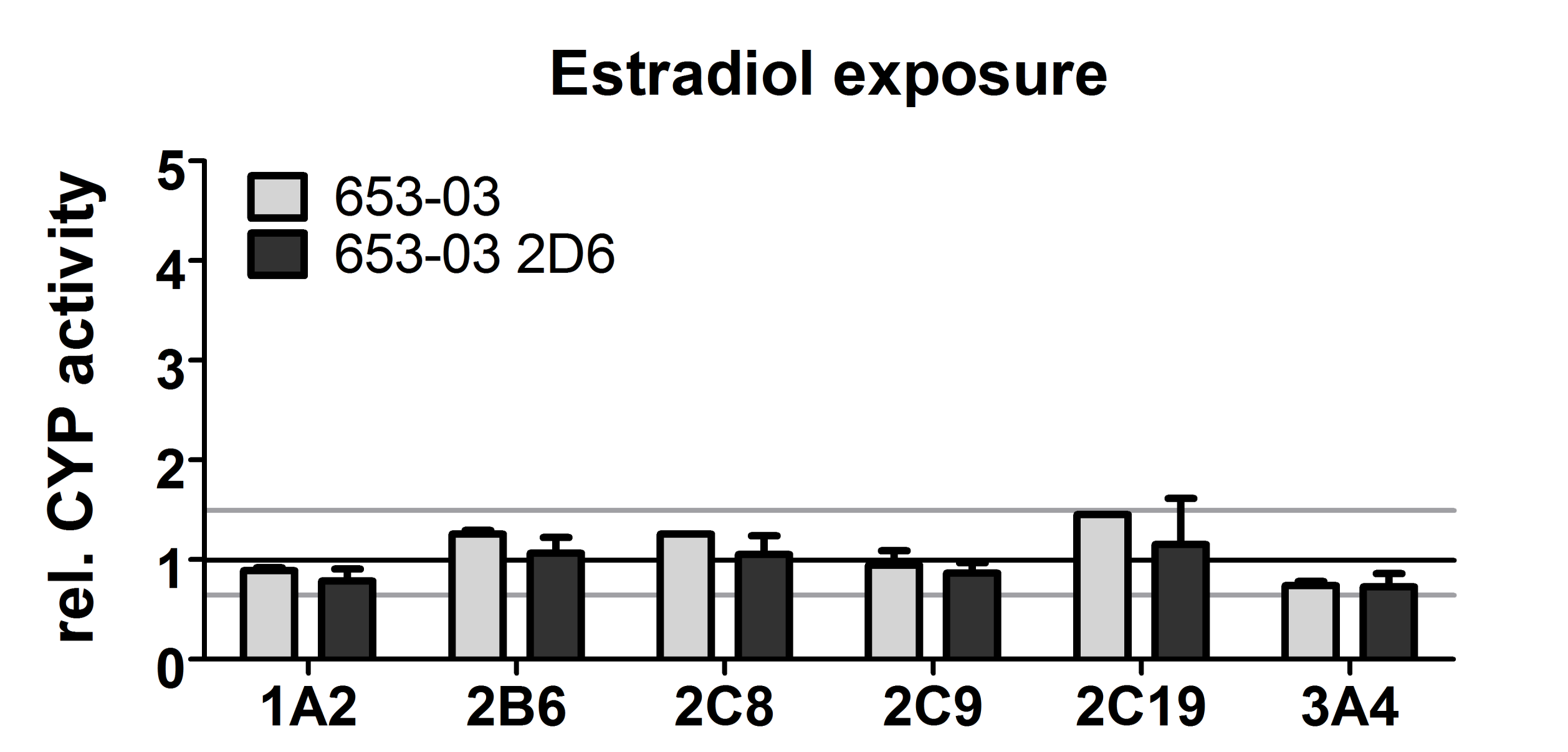

Supplement: Supplementary file 4 — Supplementary material 4 (PNG 119 KB) [file 204_2017_2147_MOESM4_ESM.png]
